# Delayed Onset Immune-Related Pituitary Adrenal Insufficiency Induced by Neoadjuvant Nivolumab Therapy for Locally Advanced Lung Cancer in the Postoperative Period: A Case Report

**DOI:** 10.70352/scrj.cr.24-0044

**Published:** 2025-02-01

**Authors:** Takafumi Kabuto, Shizuka Kaneko, Shinnosuke Nomura, Satoshi Terashita, Kaito Kitahori, Masaki Ikeda, Naohisa Chiba, Masashi Ishikawa

**Affiliations:** 1 Department of Thoracic Surgery, Japanese Red Cross Wakayama Medical Center, Wakayama, Wakayama, Japan; 2 Department of Diabetes and Endocrinology, Japanese Red Cross Wakayama Medical Center, Wakayama, Wakayama, Japan; 3 Department of Cardiovascular Medicine, Japanese Red Cross Wakayama Medical Center, Wakayama, Wakayama, Japan; 4 Department of Respiratory Medicine, Japanese Red Cross Wakayama Medical Center, Wakayama, Wakayama, Japan

**Keywords:** lung cancer, neoadjuvant therapy, immune checkpoint inhibitor, immune-related adverse event, adrenal insufficiency

## Abstract

**INTRODUCTION:**

Neoadjuvant, adjuvant, and perioperative immune checkpoint inhibitor (ICI) regimens for treating locally advanced lung cancer have dramatically evolved in recent years. Despite these immunotherapies being very promising, they can be associated with potential life-threatening immune-related adverse events (irAEs), and there is not much awareness regarding irAEs in surgical regimens.

**CASE PRESENTATION:**

A Japanese man in his 70s was diagnosed with right upper lobe lung adenocarcinoma (cT3N1[#12u]M0, parietal pleural invasion, cStage IIIA), with the programmed death-ligand 1 expression level of <1%. He underwent right upper lobectomy via open thoracotomy followed by 3 cycles of neoadjuvant cisplatin, pemetrexed, and nivolumab. The pathological response rate was 50% and the pathological stage was ypT2bN0M0, ypStage IIA. Seven months after the surgery, he experienced gradually worsening anorexia, fatigue, and hyponatremia. He was diagnosed with pituitary adrenal insufficiency induced by neoadjuvant immunotherapy by the 100 μg corticotropin-releasing hormone stress test. Cardiogenic shock caused by takotsubo cardiomyopathy occurred, and intensive treatment was performed. Steroid therapy was effective, but the physical dysfunction persisted, although no recurrence of lung cancer was observed.

**CONCLUSIONS:**

Patients receiving neoadjuvant immunotherapies can develop life-threatening irAEs late in the postoperative period. Surgeons who follow up patients after neoadjuvant immunotherapies must be as vigilant regarding the development of irAEs in the postoperative phase as clinical oncologists.

## Abbreviations

ICI
: immune checkpoint inhibitor
irAE
: immune-related adverse effect
ACTH
: adrenocorticotropic hormone
ICU
: intensive care unit

## INTRODUCTION

Surgical resection followed by adjuvant chemotherapy is recommended for locally advanced lung cancer. However, the postoperative outcomes are unsatisfactory. Neoadjuvant, adjuvant, and perioperative immune checkpoint inhibitor (ICI) regimens have recently been investigated in clinical trials for locally advanced lung cancer and have been reported to be promising and safe.^1–3)^ However, the development of immune-related adverse events (irAEs), which is the unique toxicity of ICIs, in surgical settings remains unclear and needs to be carefully monitored. We hereby present a case of severe pituitary adrenal insufficiency induced by the combination of neoadjuvant chemotherapy and nivolumab, which is an anti-programmed death protein 1 inhibitor used for treating lung adenocarcinoma in the postoperative period.

## CASE PRESENTATION

**Fig. 1:**
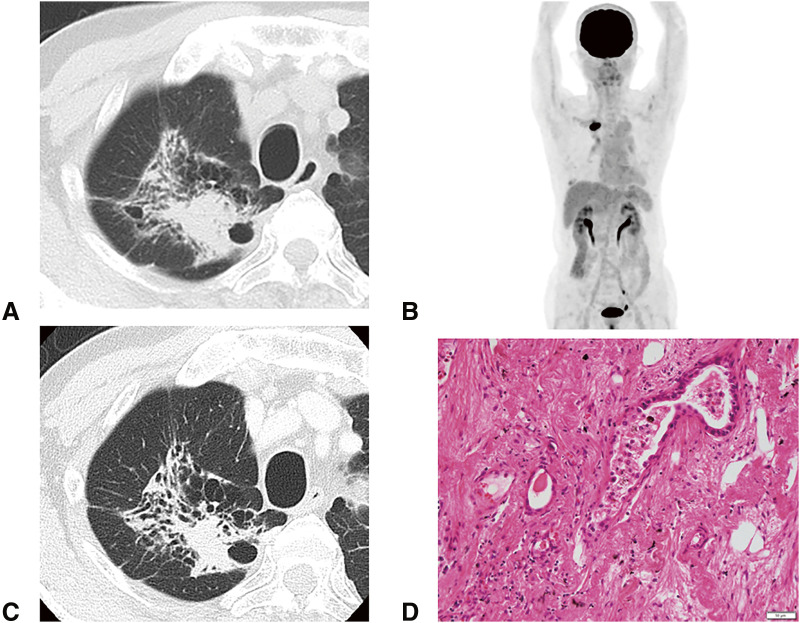
Patient’s clinicopathological findings. (**A**) and (**B**) Images obtained with CT and fluorodeoxyglucose-positron emission tomography at the first examination, respectively. (**C**) CT image was obtained after 3 cycles of neoadjuvant cisplatin, pemetrexed, and nivolumab. (**D**) Results of hematoxylin and eosin staining (×20) of the specimen. The malignant cells are damaged. CT: computed tomography

**Fig. 2:**
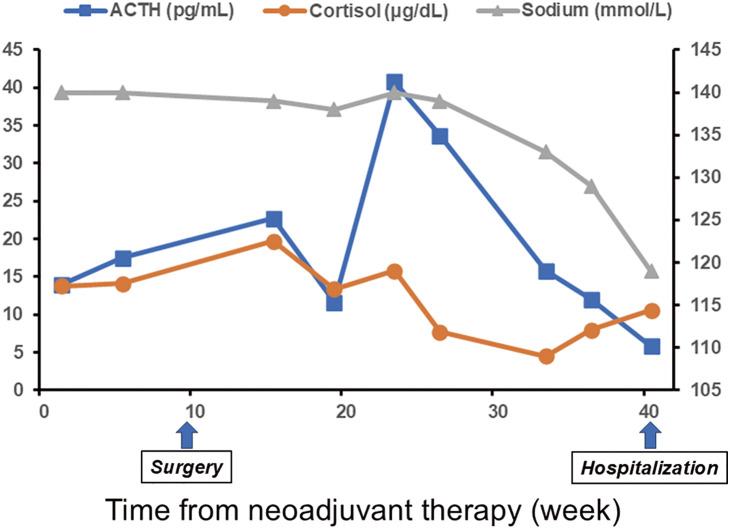
Transition of ACTH, cortisol, and serum sodium levels from the neoadjuvant therapy. ACTH and cortisol values are depicted on the left axis, and serum sodium on the right axis. ACTH: adrenocorticotropic hormone

**Fig. 3:**
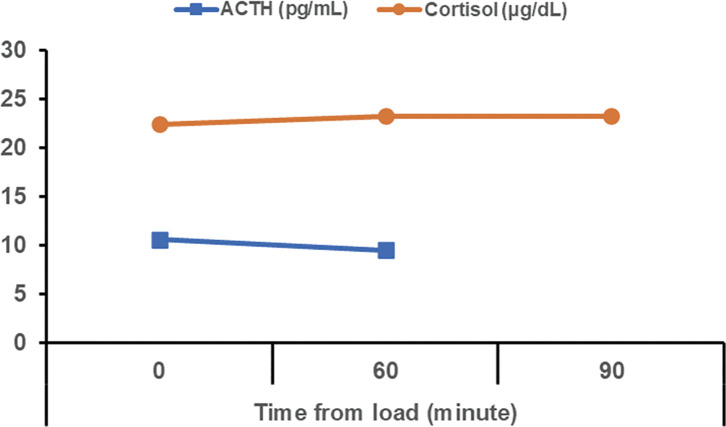
Corticotropin-releasing hormone stress test. No surge of ACTH and cortisol was observed. ACTH: adrenocorticotropic hormone

A Japanese man in his 70s was diagnosed with right upper lobe adenocarcinoma (cT3N1[#12u]M0, parietal pleural invasion, cStage IIIA), with the programmed death-ligand 1 (PD-L1) expression level of <1% (**Fig. 1A** and **1B**). The driver gene mutation was positive for mesenchymal–epithelial transformation gene exon 14 skipping and negative for epidermal growth factor receptor and anaplastic lymphoma kinase. The joint conference adopted the strategy of surgical resection following neoadjuvant immunotherapy. He received 3 cycles of neoadjuvant cisplatin, pemetrexed, and nivolumab. No significant adverse events were observed. Computed tomography images showed no significant change in the tumor after the neoadjuvant therapy (**Fig. 1C**). He then underwent a right upper lobectomy via open thoracotomy 4 weeks following the last dose of treatment. The postoperative course was uneventful except for prolonged air leakage, which was treated with autologous blood pleurodesis. The tumor cells were damaged (**Fig. 1D**), and there were inflammatory scars in the parietal pleura, which indicated a past parietal pleural invasion. The pathological response rate was 50% and the pathological stage was ypT2bN0M0, ypStage IIA. After surgery, he experienced gradually worsening anorexia and fatigue. A routine laboratory examination for irAE initially revealed no abnormal findings. However, 7 months after the surgery, the levels of both adrenocorticotropic hormone (ACTH) and cortisol decreased, and he had to be hospitalized when his serum sodium level was 119 mEq/L (**Fig. 2**). His body weight had decreased from 61 kg to 43 kg 9 months following the first administration of neoadjuvant therapy. Urinary sodium level at this point was 77 mEq/L, indicating an absence of sodium reabsorption in the tubules despite low serum sodium levels. The 100 μg corticotropin-releasing hormone stress test and radiological imaging demonstrated that there was no increase in the levels of ACTH and no abnormalities in the pituitary gland (**Fig. 3**). He was therefore diagnosed with pituitary adrenal insufficiency, in the form of an irAE. While the treatment plan was being discussed, he experienced sudden onset cardiogenic shock. Left ventriculography revealed takotsubo cardiomyopathy, and he had to be transferred to the intensive care unit (ICU). Methylprednisolone pulse therapy (1 g/day, 3 days) started because the cardiomyopathy was probably also an irAE. The steroid treatment was successful, and he was discharged from the ICU in 4 days. The methylprednisolone was subsequently switched to prednisolone, which was tapered off in 2 months from the administration while continuously monitoring the patient’s symptoms. Additionally, hormone replacement therapy in the form of hydrocortisone (15 mg in the morning and 5 mg in the evening each day) was continued. His anorexia and malnutrition persisted, and his level of activities of daily living continued to decline without any recurrence of the lung cancer. He was transferred for rehabilitation 2 months following the hospitalization.

## DISCUSSION

ICIs are one of the standard treatments for systemic lung cancer. Following the trend, neoadjuvant, adjuvant, and perioperative immunotherapy regimens have been recently approved for treating locally advanced lung cancer and are expected to gain popularity in real-world clinical practice.^1–3)^ We selected the CheckMate 816 regimen for this patient because the efficacy of the neoadjuvant regimen was proven, especially for cStage IIIA, and the pathological complete response rate of neoadjuvant nivolumab plus chemotherapy was higher than that of chemotherapy alone regardless of disease stage and the PD-L1 expression level.^1)^ This report was the first to describe the detailed clinical course of irAEs in the form of adrenal insufficiency in the late postoperative period caused by the neoadjuvant ICI regimen. This case can increase the awareness of surgeons, particularly, those who are responsible for treating patients after neoadjuvant ICI therapy but are unfamiliar with the irAEs.

The overall incidence rate of endocrinological irAEs is approximately 10%, whereas that of Grade 3 or higher is less than 1%.^4)^ The immune-mediated pituitary adverse events are related to favorable prognosis.^5)^ Actually, there were no incidents of recurrent lesions of lung cancer in the current case. The incidence of irAEs in surgery-related ICI regimens in clinical trials has been considered rare and acceptable.^1–3)^ For example, the treatment regimen for the CheckMate 816 trial that was decided for the particular patient was associated with a 1.1% incidence rate of Grade 3 or 4 adrenal insufficiency as an irAE, but the observation covered less than 100 days from the last dose.^1)^ Furthermore, there were no incidences of cardiac adverse events in the trial. The fact that takotsubo cardiomyopathy observed in this case is an irAE can be controversial since takotsubo cardiomyopathy can occur secondary to adrenal insufficiency, and the incidence of ICI-induced myocarditis is commonly observed within 12 weeks following an ICI therapy.^6,7)^ Irrespective of whether the etiology is immune-mediated, it should be considered a severe cardiac adverse event of the ICI regimen.

We need to understand that in the real world, irAE cases can occur several months following the end of ICI administration.^8)^ In the present case, the patient complained of symptoms of anorexia and fatigue following the surgery, regardless of the normal level of ACTH and cortisol. Abnormal findings that raised suspicions of adrenal insufficiency were observed several months later. Moreover, his symptoms, which were nonspecific and mimicked prolonged postoperative fatigue, could have masked the endocrine abnormalities. Therefore, making the diagnosis of irAE was difficult. Reduced levels of serum cortisol accompanied by abnormal ACTH levels or the presence of mild hyponatremia compared to the baseline could be the signs of adrenal insufficiency^5)^; for example, one point prior to hospitalization in **Fig. 2** might be an earlier chance of detecting the endocrinological abnormalities in this case. Although, currently, no consensus is available regarding the end of irAE follow-up following ICI cessation, irAEs must be diagnosed and managed appropriately when patients who have a history of ICI treatment complain of poor physical condition.

## CONCLUSIONS

Patients receiving neoadjuvant immunotherapies can develop life-threatening irAEs late in the postoperative period. The diagnosis can be difficult because the symptoms are sometimes nonspecific. If patients complain of any symptoms after completion of neoadjuvant immunotherapy, irAE should be suspected with caution. Early detection, follow-up, and management of irAEs are necessary not only for clinical oncologists but also for surgeons.

## DECLARATIONS

### Funding

There is no funding to report for this submission.

### Authors’ contributions

Takafumi Kabuto was mainly in charge of the surgery, follow-up, and management of irAE of the patient and wrote the original draft.

Shizuka Kaneko and Shinnosuke Nomura treated the endocrinological and cardiological disorders, respectively.

Satoshi Terashita was in charge of the neoadjuvant treatment.

Kaito Kitahori, Masaki Ikeda, Naohisa Chiba, and Masashi Ishikawa assisted with the patient’s treatment.

All the authors have read and approved the final version of the manuscript.

### Availability of data and materials

Data will be made available from the corresponding author upon reasonable request.

### Ethics approval and consent to participate

This work does not require ethical considerations or approval. Informed consent was obtained from the patient for using the patient’s clinical data and the accompanying images.

### Consent for publication

Informed consent was obtained from the patient for publication of the patient’s clinical data and the accompanying images.

### Competing interests

The authors declare that they have no competing interests.
